# Non-aneurysmal Subarachnoid Hemorrhage Presenting With Isolated Neck and Back Pain: A Rare Case Report

**DOI:** 10.7759/cureus.94422

**Published:** 2025-10-12

**Authors:** Binyam M Habte, Yoseph M Habte, Biruk W Fantu, Makida M Habte, Esimael M Abdu

**Affiliations:** 1 Department of Medicine, ALERT Comprehensive Specialized Hospital, Addis Ababa, ETH; 2 Department of Medicine, Ethio Tebib Hospital, Addis Ababa, ETH; 3 Department of Medicine, Axon Stroke and Spine Centre, Addis Ababa, ETH; 4 Department of Medicine, Bethel Medical College, Addis Ababa, ETH; 5 Department of Surgery, Teklehaimanot General Hospital, Addis Ababa, ETH

**Keywords:** atypical presentation, case report, digital subtraction angiography, non-aneurysmal subarachnoid hemorrhage, spinal pain in subarachnoid hemorrhage

## Abstract

Subarachnoid hemorrhage (SAH) is defined as bleeding into the subarachnoid space between the arachnoid and pia mater and is most commonly caused by rupture of an intracranial aneurysm if it occurs spontaneously. SAH typically presents with sudden, severe headache, often accompanied by nausea, vomiting, and neck stiffness. We report a rare case of non-aneurysmal SAH (NASAH) in an 83-year-old woman presenting with isolated acute neck pain and worsening chronic back pain, without headache or neurological deficits. Laboratory evaluation revealed hemorrhagic cerebrospinal fluid. Head and neck MRI revealed degenerative cervical spine changes and localized subarachnoid hemorrhage in the premedullary and cerebellomedullary cisterns, with no aneurysm or vascular abnormality detected on magnetic resonance angiography (MRA) and digital subtraction angiography (DSA). The patient was managed conservatively with neurological and hemodynamic monitoring, blood pressure control, analgesia, and supportive care. Follow-up imaging showed partial resolution of the hemorrhage, and she remained neurologically intact. This case highlights the importance of considering SAH in atypical presentations to ensure timely diagnosis and management.

## Introduction

Spontaneous subarachnoid hemorrhage (SAH) is bleeding into the subarachnoid space, most commonly caused by rupture of an intracranial aneurysm, and represents a neurological emergency with high morbidity and mortality [1]. Classically, SAH presents with sudden, severe headache, often accompanied by nausea, vomiting, and neck stiffness [2]. However, we report a rare case of non-aneurysmal SAH (NASAH) in an 83-year-old woman who presented solely with acute neck pain and worsening of chronic back pain. This atypical presentation underscores the importance of maintaining a high index of suspicion and the role of prompt neuroimaging, particularly in elderly patients with comorbidities, to ensure timely diagnosis and appropriate management.

## Case presentation

An 83-year-old woman with a known history of type 2 diabetes mellitus, diagnosed four years earlier, and hypertension, diagnosed two years earlier, presented with a complaint of worsening neck and back pain for five days. Her regular medications included metformin, lisinopril, metoprolol succinate, clopidogrel, pregabalin, and gabapentin.

She reported a history of intermittent lower back pain over the past four years, radiating to both lower limbs. In the five days prior to her presentation, the pain worsened significantly and was accompanied by new-onset neck pain. The neck pain was described as a constant, dull aching localized to the posterior cervical region without radiation. This was also associated with two episodes of non-projectile vomiting of ingested matter. She denied headache, photophobia, seizures, focal neurological deficits, sensory loss, or changes in consciousness. Her past medical history was notable for cerebral vascular narrowing, diagnosed five years earlier following an episode of dizziness and syncope. She was initially treated with aspirin but was later switched to clopidogrel due to an allergy to aspirin.

On examination, she was alert, oriented, and hemodynamically stable with a blood pressure of 165/90 mmHg, heart rate 78 beats per minute, respiratory rate 18 breaths per minute, temperature 36.7°C, and oxygen saturation 97% on room air. General physical examination was unremarkable except for neck and paraspinal tenderness. Neurological examination revealed no cranial nerve deficits, motor weakness, sensory loss, or reflex asymmetry. Meningeal signs (Kernig’s and Brudzinski’s) were negative.

Initial laboratory investigations, including complete blood count, renal and liver function tests, and coagulation profile, were all within normal limits, indicating no evidence of systemic infection, organ dysfunction, or coagulopathy. Given her persistent neck and back pain with associated vomiting, in the absence of an alternative explanation on examination and basic labs, a lumbar puncture was performed to exclude infectious etiologies. Cerebrospinal fluid (CSF) analysis showed a normal opening pressure, with uniformly hemorrhagic fluid across multiple collection tubes, and xanthochromia observed upon centrifugation, consistent with SAH rather than a traumatic tap (Table 1).

**Table 1:** Laboratory investigations with corresponding results and reference values. All values were within normal reference range except for cerebrospinal fluid (CSF) analysis which revealed hemorrhagic fluid with elevated red blood cell count and xantochromia, consistent with subarachnoid hemorrhage.

Laboratory Parameters	Results	Normal Value
Complete Blood Count		
White Blood Cell	10.3 × 10^3^/µL	4.0 - 11.0 × 10^3^/µL
Hemoglobin	13.8 g/dL	13.5 - 17.5 g/dL
Platelet	154 × 10^3^/µL	150 - 450 × 10^3^/µL
Lymphocyte percentage	25.3%	15 - 50%
Neutrophil percentage	70.1%	45 - 80%
Metabolic Panel		
Creatinine	1.1 mg/dl	0.67 - 1.17 mg/dl
Urea	36.3 mg/dl	17- 43 mg/dl
Na+	139 mmol/l	136 - 145 mmol/l
K+	3.6 mmol/l	3.5 - 5.1 mmol/l
Aspartate Transaminase	38.6 U/L	2 - 50 U/L
Alanine Transaminase	36.3 U/L	1 - 50 U/L
Uric Acid	5.77 mg/dL	2.4 – 6.0 mg/dL
Coagulation Profile		
Prothrombin Time	12.3 seconds	10.7 - 14.3 seconds
International Normalized Ratio	1.0	0.8 - 1.2
Activated Partial Thromboplastin Time	22.1 seconds	21 - 35 seconds
Serology		
Vitamin B12	708 pmol/L	204-831 pmol/L
Vitamin D	42 ng/mL	30 – 100 ng/mL
Thyroid Stimulating hormone	3.8 mIU/L	0.4 - 4.0 mIU/L
Venereal Disease Research Laboratory Test (VDRL)	Non - reactive	Non - reactive
Rheumatoid Factor qualitative	Non - reactive	Non - reactive
CSF Analysis		
Appearance	Hemorrhagic	Clear, colorless
Red Blood Cells	Numerous	0 cells/µL
Glucose	66 mg/dL	45–80 mg/dL
Protein	98 mg/dL	15–45 mg/dL
Xanthochromia	present	Absent

**Figure 1:**
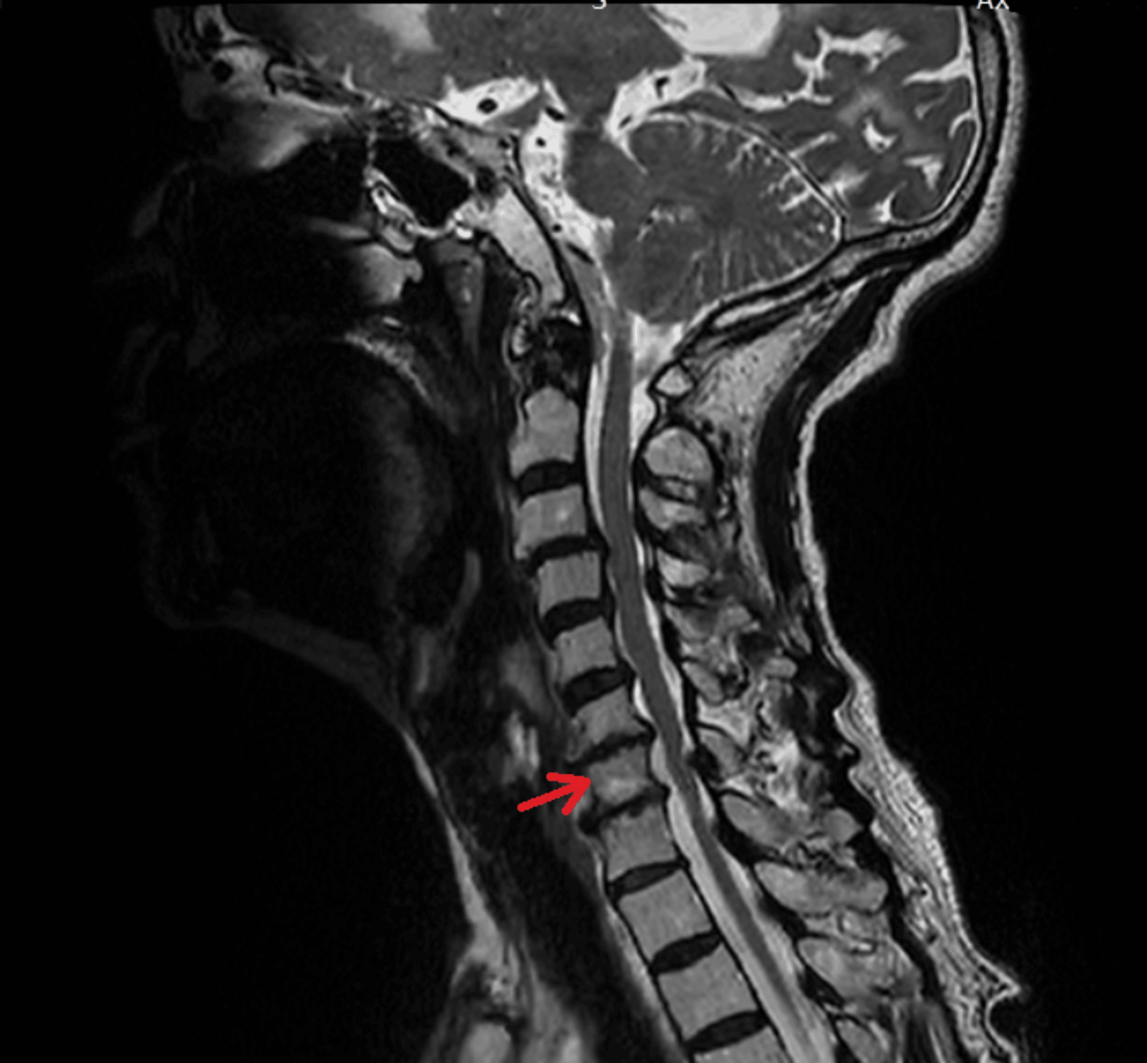
Cervical spine MRI demonstrating multilevel degenerative changes Sagittal T2-weighted MRI showing multilevel degenerative changes and mild to moderate spinal canal stenosis from C4 to C7

Given the patient’s atypical presentation, characterized by prominent neck and back pain without headache or focal neurological deficits, a cervical spine MRI was obtained to evaluate for possible spinal pathology, including structural causes of pain or a potential spinal source of hemorrhage. The imaging revealed multilevel degenerative changes, including disc dehydration and posterior disc protrusions with additional mild to moderate spinal canal stenoses from C4 to C7. No evidence of spinal cord compression or vascular malformations was identified (Figure 1).

**Figure 2:**
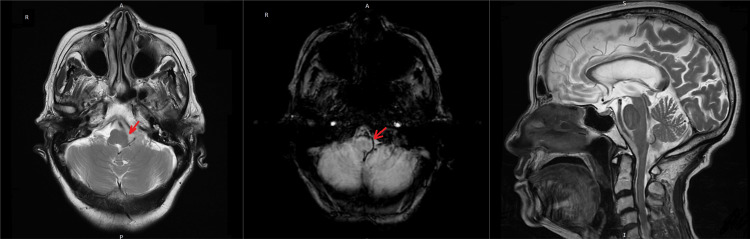
Brain MRI demonstrating subarachnoid hemorrhage Left: Axial T2-weighted MRI showing intermediate signal collection in the premedullary and left lateral cerebellomedullary cisterns (Red arrow). Middle: Axial susceptibility-weighted imaging (SWI) sequence demonstrating blooming artifacts consistent with subarachnoid hemorrhage (Red arrow). Right: Sagittal T2-weighted MRI showing the structural anatomy

In view of the hemorrhagic cerebrospinal fluid findings and to further evaluate for intracranial sources of bleeding, a brain MRI was performed. It demonstrated a T2 intermediate signal collection in the premedullary and left lateral cerebellomedullary cisterns with blooming on susceptibility-weighted imaging (SWI), consistent with SAH. Additionally, very small hemorrhages were noted in the atria of the lateral ventricles (Figure 2).

**Figure 3:**
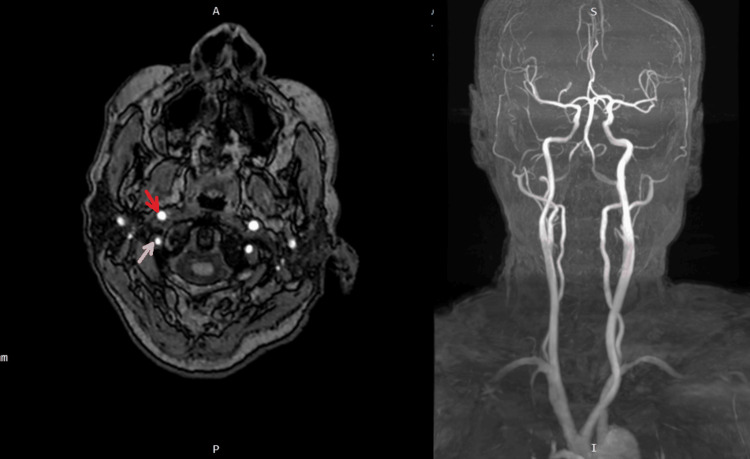
Magnetic resonance angiography (MRA) of the head and neck Left: Axial time-of-flight (TOF) MRA showing mild stenotic segments in the carotid and vertebrobasilar systems. The red arrow (internal carotid artery) and white arrow (vertebral artery) indicate the affected vessels. Right: 3D reconstructed MRA demonstrating overall vasculature without evidence of aneurysm, filling defect, or significant stenosis.

To further investigate the etiology of the hemorrhage, a magnetic resonance angiography (MRA) of the head and neck was subsequently performed. It revealed atherosclerotic changes with mild stenotic segments in the carotid and vertebrobasilar systems; however, no aneurysm, filling defect, or significant stenosis was identified. The anterior and posterior circulation vessels were otherwise unremarkable (Figure 3).

In light of the inconclusive MRA findings and the need to definitively exclude a vascular lesion, digital subtraction angiography (DSA) was subsequently performed. This revealed no evidence of intracranial aneurysm, arteriovenous malformation, or dural arteriovenous fistula. The overall impression was that of a NASAH, and further evaluation for alternative etiologies, including coagulopathy, was recommended.

The patient was admitted to the intensive care unit (ICU) for close neurological and hemodynamic monitoring, with management focused on stabilization, symptom control, and prevention of complications. Blood pressure was maintained below 160 mmHg to minimize the risk of rebleeding while preserving adequate cerebral perfusion, using her regular antihypertensive regimen of amlodipine, lisinopril, and metoprolol. Analgesia for acute pain included paracetamol and intermittent use of morphine or fentanyl as needed, while pregabalin and gabapentin were continued for chronic neuropathic pain. Lactulose was administered to prevent opioid-induced constipation. Clopidogrel was continued following multidisciplinary review, in light of her history of cerebrovascular disease. Seizure prophylaxis with levetiracetam was initiated early in the admission and later discontinued once the risk was reassessed. Magnesium sulfate was administered intravenously as part of supportive neurocritical care. Additional supportive measures included hydration, early mobilization, and gastrointestinal protection with omeprazole. Serial neurological examinations were performed, and the patient was closely monitored for potential complications, including cerebral vasospasm, hydrocephalus, and thromboembolic events. She remained in the ICU for 72 hours before being transferred to the ward once stable.

A follow-up brain MRI obtained prior to discharge demonstrated partial resolution of the subarachnoid hemorrhage with no evidence of rebleeding. The patient remained neurologically stable and was discharged with advice for strict blood pressure control, ongoing imaging surveillance, and regular neurology follow-up.

## Discussion

SAH is defined as bleeding into the subarachnoid space between the arachnoid and pia mater, most commonly resulting from rupture of an intracranial aneurysm [1]. It is a neurological emergency associated with significant morbidity and mortality. However, in around 15% of cases, no vascular lesion is detected despite DSA [3]. These cases are classified as NASAH and are further subdivided into perimesencephalic SAH (PMN-SAH) and non-perimesencephalic SAH (nPMN-SAH) based on the distribution of blood. This distinction is clinically important, as PMN-SAH carries an excellent prognosis with a very low risk of rebleeding or delayed cerebral ischemia, whereas nPMN-SAH is associated with higher complication rates and may occasionally unmask small aneurysms on repeat angiography [4].

PMN-SAH was first described by van Gijn et al. as a benign entity, characterized by the distribution of the subarachnoid hemorrhage mainly or only in the perimesencephalic cisterns [5]. Radiologically, it is characterized by several key features: blood centered anterior to the midbrain in the interpeduncular, prepontine, or premedullary cisterns; minimal extension into the ambient cisterns or proximal Sylvian fissures without diffuse spread into the interhemispheric fissures, convexities, or distal Sylvian fissures; no or only minimal intraventricular blood; absence of parenchymal hematoma; and absence of aneurysm or vascular malformation on high-quality angiography, with DSA considered the gold standard [6]. Our patient’s hemorrhage fulfilled these criteria: the bleeding was localized to the premedullary and cerebellomedullary cisterns, with only minimal intraventricular extension, no parenchymal involvement, and no vascular lesion on both MRA and confirmatory DSA. This pattern, together with her benign clinical course and radiological resolution on follow-up imaging, supports the diagnosis of perimesencephalic non-aneurysmal SAH.

Clinically, patients with SAH most often present with the abrupt onset of a severe headache, frequently described as “the worst headache of their life.” This classical presentation is often accompanied by meningeal irritation signs such as neck stiffness, photophobia, nausea, and vomiting. Compared with aneurysmal SAH, focal neurological deficits, seizures, and depressed consciousness are far less common in non-aneurysmal SAH, and many patients remain neurologically intact throughout their course [2]. However, our patient represents an atypical presentation: she did not experience headache at any point but instead presented with acute-onset neck pain and worsening back pain. This unusual symptomatology may be explained by localized meningeal irritation from cisternal blood in the posterior fossa and upper spinal subarachnoid spaces, without the diffuse supratentorial irritation that typically produces thunderclap headache [7]. Such atypical presentations are rarely reported and highlight the importance of considering SAH in patients with acute neck or back pain, especially when accompanied by nuchal and paraspinal tenderness.

When evaluating SAH with blood localized around the brainstem cisterns, alternative etiologies should also be considered, including small ruptured posterior circulation aneurysms (particularly basilar tip or posterior inferior cerebellar artery (PICA)), arteriovenous malformations or dural fistulae, cavernous malformations with cisternal rupture, and hemorrhagic neoplasms or trauma [8]. High-resolution vascular imaging is therefore mandatory to exclude these entities. In this case, the absence of any vascular abnormality on repeated angiographic studies and the classic perimesencephalic distribution made these differentials unlikely. The diagnostic pathway for suspected SAH begins with non-contrast CT, which is highly sensitive within the first six hours but decreases in yield thereafter. When CT is negative but suspicion remains high, lumbar puncture demonstrating xanthochromia or persistently hemorrhagic CSF establishes the diagnosis [9]. MRI with fluid-attenuated inversion recovery (FLAIR) and SWI sequences can detect small or delayed bleeds, as in our patient [10]. Once hemorrhage is confirmed, vascular imaging is essential: CTA and MRA provide rapid non-invasive screening, but DSA remains the gold standard to definitively exclude aneurysms, particularly in nPMN-SAH [11]. In PMN-SAH, repeat angiography has a very low diagnostic yield and is not routinely recommended after a high-quality negative DSA [8].

The precise origin of PMN-SAH remains debated. The leading hypothesis suggests a venous source, particularly rupture of veins in the perimesencephalic region such as the basal vein of Rosenthal, given the limited blood distribution, mild clinical course, and lack of rebleeding [12,13]. The venous hypothesis best explains the localized bleeding pattern and the benign outcome observed in our patient.

Management of PMN-SAH is largely supportive, aimed at controlling blood pressure, monitoring for hydrocephalus, and providing symptomatic relief due to the lower risk of rebleeding and better prognosis. Unlike aneurysmal SAH, prophylactic interventions against rebleeding, such as securing the aneurysm by surgical clipping or endovascular coiling, are not required [3]. Our patient was admitted for close neurological and hemodynamic observation. Blood pressure was stabilized with antihypertensives, analgesia was optimized, and she was monitored for complications such as vasospasm and hydrocephalus, neither of which developed. Importantly, her course remained benign, and follow-up MRI demonstrated partial resolution of the hemorrhage. She was discharged neurologically intact with arrangements for ongoing surveillance.

This case underscores that not all patients with subarachnoid hemorrhage present with the classic thunderclap headache. Our patient’s isolated neck and back pain, without headache or neurological deficits, represents an atypical manifestation that could easily be mistaken for degenerative spine disease, particularly in the elderly. Such cases highlight the importance of maintaining vigilance for SAH even in atypical presentations, especially among patients with risk factors such as hypertension, diabetes, or antithrombotic therapy. Prompt neuroimaging and angiographic evaluation are essential to exclude aneurysmal disease, while careful interpretation is necessary to avoid unnecessary interventions in these patients.

## Conclusions

This case demonstrates that subarachnoid hemorrhage can present atypically, without the classic thunderclap headache, and manifest solely as isolated neck and back pain. Such unusual presentations are easily overlooked, particularly in elderly patients with comorbidities or chronic musculoskeletal pain. Clinicians should maintain a high index of suspicion and pursue timely neuroimaging and angiographic evaluation to ensure accurate diagnosis, guide appropriate management, and prevent potentially serious complications.
